# Next generation flow for minimally-invasive blood characterization of MGUS and multiple myeloma at diagnosis based on circulating tumor plasma cells (CTPC)

**DOI:** 10.1038/s41408-018-0153-9

**Published:** 2018-11-19

**Authors:** L. Sanoja-Flores, J. Flores-Montero, J. J. Garcés, B. Paiva, N. Puig, A. García-Mateo, O. García-Sánchez, A. Corral-Mateos, L. Burgos, E. Blanco, J. Hernández-Martín, R. Pontes, M. Díez-Campelo, P. Millacoy, P. Rodríguez-Otero, F. Prosper, J. Merino, M. B. Vidriales, R. García-Sanz, A. Romero, L. Palomera, R. Ríos-Tamayo, M. Pérez-Andrés, J. F. Blanco, M. González, J. J. M. van Dongen, B. Durie, M. V. Mateos, J. San-Miguel, A. Orfao, on behalf of the EuroFlow consortium

**Affiliations:** 1 Cancer Research Center (IBMCC-CSIC/USAL-IBSAL); Cytometry Service (NUCLEUS) and Department of Medicine, University of Salamanca (USAL), Salamanca, Spain; 2 Centro de Investigación Biomédica en Red de Cáncer: CIBER-ONC number CB16/12/00400, Instituto Carlos III, Madrid, Spain; 3 Clinica Universidad de Navarra (UNAV), Applied Medical Research Center (CIMA), IDISNA. CIBER-ONC number CB16/12/00369 and CB16/12/00489, Pamplona, Spain; 4 Department of Hematology, University Hospital of Salamanca (HUSA), IBSAL; IBMCC (USAL-CSIC). CIBER-ONC number CB16/12/00233, Salamanca, Spain; 5 Department of Hematology, Health Care Center of Segovia (CAS), Segovia, Spain; 6 Faculty of Medicine, Federal University of Rio de Janeiro and Institute of Pediatrics and Childhood Care, Rio de Janeiro, Brazil; 7 Department of Hematology, Hospital Center of Navarra (CHN), Pamplona, Spain; 8 Primary Care Center Miguel Armijo, Sanidad de Castilla y León (SACYL), Salamanca, Spain; 9 Department of Hematology, University Hospital Lozano Blesa (HULB), Zaragoza, Spain; 10 Department of Hematology, Virgen de las Nieves Hospital (HVN), Granada, Spain; 11 Department of Orthopedics, University Hospital of Salamanca, IBSAL; IBMCC (USAL-CSIC), Salamanca, Spain; 12 Department of Immunohematology and Blood Transfusion, Leiden University Medical Center, Leiden, The Netherlands; 13 Cedars-Sinai Samuel Oschin Cancer Center, Los Angeles, CA USA

## Abstract

Here, we investigated for the first time the frequency and number of circulating tumor plasma cells (CTPC) in peripheral blood (PB) of newly diagnosed patients with localized and systemic plasma cell neoplasms (PCN) using next-generation flow cytometry (NGF) and correlated our findings with the distinct diagnostic and prognostic categories of the disease. Overall, 508 samples from 264 newly diagnosed PCN patients, were studied. CTPC were detected in PB of all active multiple myeloma (MM; 100%), and smoldering MM (SMM) patients (100%), and in more than half (59%) monoclonal gammopathy of undetermined significance (MGUS) cases (*p* <0.0001); in contrast, CTPC were present in a small fraction of solitary plasmacytoma patients (18%). Higher numbers of CTPC in PB were associated with higher levels of BM infiltration and more adverse prognostic features, together with shorter time to progression from MGUS to MM (*p* <0.0001) and a shorter survival in MM patients with active disease requiring treatment (*p* ≤ 0.03). In summary, the presence of CTPC in PB as assessed by NGF at diagnosis, emerges as a hallmark of disseminated PCN, higher numbers of PB CTPC being strongly associated with a malignant disease behavior and a poorer outcome of both MGUS and MM.

## Introduction

Plasma cell neoplasms (PCN) are a heterogeneous group of diseases characterized by the clonal expansion of terminally-differentiated plasma cells (PC)^1–4^. Whereas monoclonal gammopathy of undetermined significance (MGUS) and smoldering multiple myeloma (SMM) represent pre-malignant phases of the disease with progressively higher degree of bone marrow (BM) involvement and relatively low rates of malignant transformation (i.e., 1 and 10% per year, respectively^5–7^), multiple myeloma (MM) is an active malignancy usually associated with end-organ damage requiring therapy, and potential for transformation into PC leukemia (PCL)^1,8,9^. In turn, solitary plasmacytoma (SP) consists of a localized accumulation of tumor (mono) clonal PC (TPC) in a specific tissue area, without evidence for systemic disease^10,11^, but a rate of transformation to MM of ~ 15%–50%, depending on the primary localization of the tumor (e.g., soft-tissue vs. bone plasmacytoma, respectively)^12,13^.

Despite BM is the most frequently involved tissue in PCN^9,14,15^, and a close interaction with the BM microenvironment is required for long-term persistence of normal plasma cells (NPC) and TPC^16–18^, previous studies have recurrently shown involvement of peripheral blood (PB) in a substantial fraction of patients^15,19–24^. However, the frequency of PB involvement depends on the sensitivity of the methods used and the specific diagnostic subtype of PCN^15,19–23^. Thus, PB involvement by circulating TPC (CTPC) increases from MGUS -19 to 37%- to MM -50 to 75%-^19,24^^–^^26^, and PCL (100%)^8,27^, depending on whether immunocytochemistry or conventional 4–8-color flow cytometry are used, respectively.

Despite such variability and the relatively low sensitivity of the methods used so far, the presence of CTPC in PB of newly diagnosed MGUS and SMM patients has been associated with an increased risk of progression to MM^20,21,25–29^, and within MM with an adverse outcome^14,30^, both when evaluated at diagnosis and after therapy^31–33^. Recently, a next-generation flow cytometry (NGF) approach has been established for high-sensitive minimal residual disease (MRD) monitoring in the BM of MM patients, after therapy^34,35^. However, no study has investigated so far whether NGF also increases the frequency of detection of very low levels of PB involvement by CTPC in newly diagnosed PCN patients, and its potential prognostic impact.

Here, we investigated for the first time the frequency and number of CTPC in PB of 264 newly diagnosed patients with localized (i.e., SP) and systemic (i.e., MGUS, SMM, and MM) PCN using NGF^34^, and correlated our findings with the distinct diagnostic and prognostic categories of the disease.

## Patients and methods

### Patients and samples

Overall, 508 samples -264 PB and 244 paired BM samples- from 264 patients (53% males and 47% females; median age of 69 years, ranging from 28 to 97 years) with newly diagnosed PCN, were studied. In parallel, 71 PB and 12 BM samples from sex- and age-matched healthy donors (HD) were also investigated. Patients were classified according to the International Myeloma Working Group (IMWG) criteria^36^ into: 150 MGUS, 97 multiple myeloma patients (72 MM and 25 SMM) and, 17 SP patients (Supplemental Table 1). Four MM patients presented with >1 focal lesion associated with multiple osteolytic lesions (CRAB criteria) but minimal BM involvement by PC on cytomorphology (percentage of BMPC of 2%, 3%, 7%, and 17%, respectively) (Supplemental Table 2); hereafter, these four cases are referred to as macrofocal MM (macrofocalMM)^37–39^. MM and SMM that progressed to MM were both uniformly treated according to the Spanish PETHEMA protocols^40,41^. Written informed consent was given by each individual prior to entering the study according to the Declaration of Helsinki, and the study was approved by the local ethics committees. All samples were received from the different participating centers (USAL/HUSA, UNAV, CAS, CHN, HULB, HVN) and centrally processed at either USAL or UNAV within 24 h after they had been collected. None of the samples received was inadequate for further staining and processing.

### Risk-stratification of MGUS, SMM, and MM patients

MGUS patients were stratified by the Mayo Clinic index^42^ into: score 0, 52 cases; score 1, 54; score 2, 40; and score 3, 3 patients. Most MGUS patients (89%) showed < 95% TPC within the overall BMPC compartment^43^. In turn, SMM patients were stratified into risk-groups by both the Mayo Clinic^42^ (score 0, 7 cases; score 1, 13; and, score 2, 5 patients) and the Spanish prognostic indices^43^ (score 0, 2 patients; score 1, 8; and, score 2, 12 cases); due to the low number of cases, we grouped them into just two groups: standard/low (score 0–1) vs. high (score 2) risk cases. Finally, MM patients were classified by the Revised International Staging System (R-ISS)^44^ into stage I (*n* = 12), stage II (*n* = 29), and stage III (*n* = 24) patients. In the remaining few cases, enough data was not available.

### Immunophenotypic studies

PB (median volume: 5.1 mL; range: 2.1–12.8 mL) and BM-aspirated (100 µL) samples were collected in tubes containing EDTA and processed using the EuroFlow bulk-lysis, surface membrane (Sm)-only and Sm-plus cytoplasmic (Cy) staining procedures^34^. Overall, ≥ 10 × 10^6^ PB and ≥ 1 × 10^6^ BM cells/tube were stained with the 2-tube/8-color EuroFlow-IMF MM MRD antibody panel, as described elsewhere^34^ (Supplemental Materials and Supplemental Table 3). Stained cells were measured in FACSCanto II flow cytometers -Becton/Dickinson Biosciences (BD), San Jose, CA- using the FASCDiva software (BD). The percentage of immunophenotypically NPC and TPC was calculated from both the whole sample cellularity and from the PB and BM PC compartments. In addition, PB absolute NPC and CTPC counts, were determined using a dual-platform approach^45^. For flow cytometry data analysis, the *Infinicyt* software (version 2.0; Cytognos SL, Salamanca, Spain) was used. Antigen expression levels were specifically evaluated for PB and BM TPC, and they were expressed as median fluorescence intensity values (MFI; arbitrary units scaled from 0 to 262,144). The limit of detection of the NGF approach used in both PB and BM was set at ≥ 20 tumor plasma cell events, following previously established criteria^34,46^.

### Statistical methods

For all statistical analyses the Statistical Package for Social Sciences (SPSS version 23; IBM, Armonk, NY) was used. To assess the statistical significance of differences observed between two or more than two groups, either the Mann–Whitney *U* (unpaired variables) or the Wilcoxon tests (paired variables), and the Kruskal–Wallis test were used, respectively. Receiver operating characteristic (ROC) curve analysis was applied to define the most accurate cutoff value to discriminate between MGUS and MM cases, based on the absolute number of CTPC in PB. Correlation studies were performed using the (two-sided) Spearman’s rho (*ρ*) for non-parametric paired data. The Kaplan–Meier method and either the (two-sided) log-rank or the post-hoc tests were used to plot and compare time to progression (TTP), progression-free survival (PFS) and overall survival (OS) curves between two or more than two groups, respectively. Progression was defined as transformation of MGUS into SMM or MM, and of SMM into MM. TTP, OS, and PFS were calculated as the time from diagnosis to disease progression, to death by any reason, and either to disease progression or death by any reason, respectively. Statistical significance was set at *p*-values < 0.05.

## Results

### Distribution of normal and tumor PC in PB

**Fig. 1:**
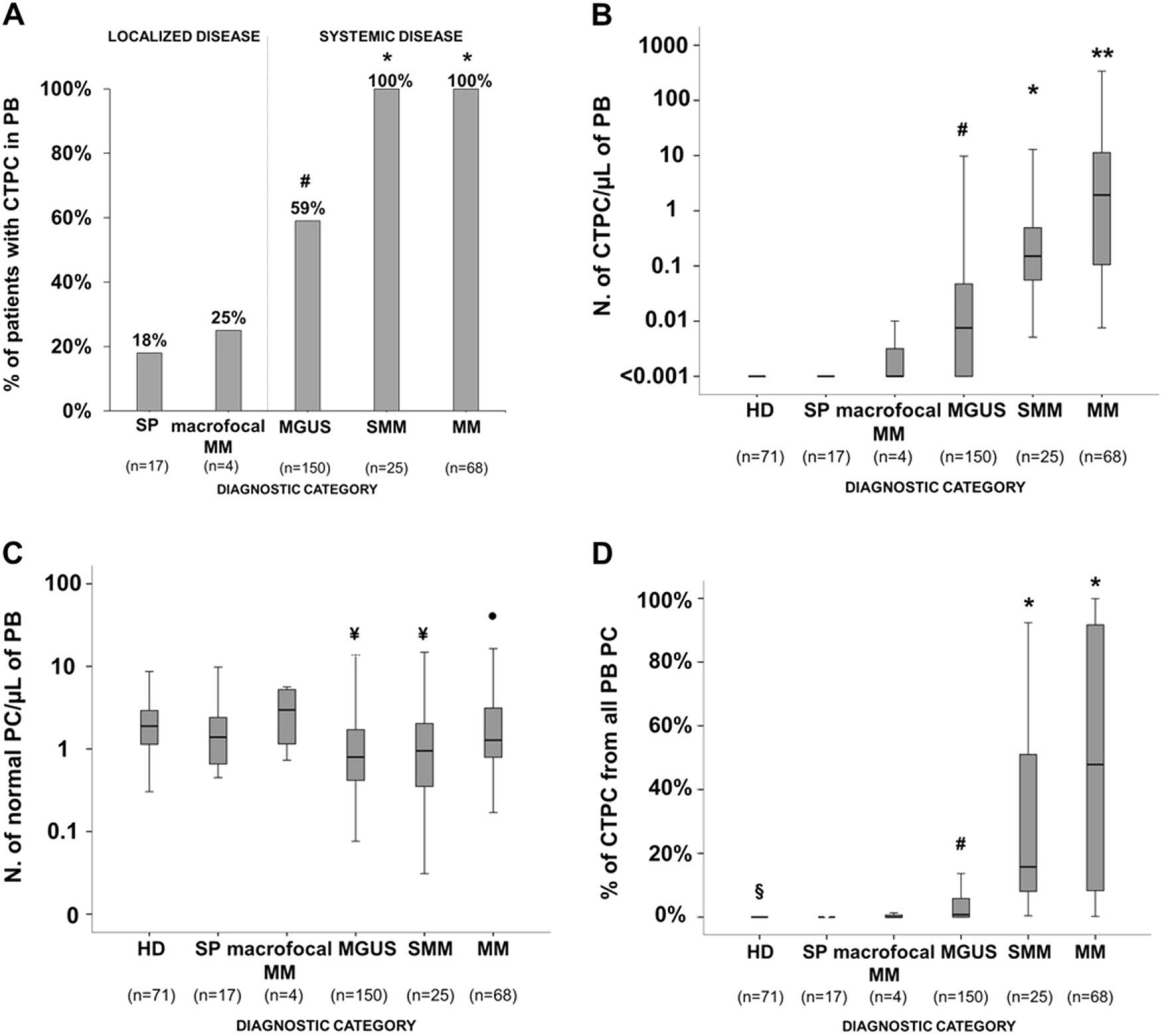
Frequency of CTPC by NGF in PB of newly diagnosed PCN patients and distribution of TPC and NPCand their ratios in HD vs PCN patients. Boxes extend from the 25th to 75th percentiles; the line in the middle and vertical lines correspond to the median value and the 10th and 90th percentiles, respectively. *p < 0.05 for SMM and MM vs. all other groups; **p < 0.05 for MM vs. all other groups; ^#^*p* < 0.05 for SP vs. MGUS; ^¥^*p* < 0.05 vs. HD; •*p* < 0.05 for MGUS vs. MM; ^§^*p* < 0.05 for HD vs. all other groups. PC plasma cell, PCN PC neoplasms, CTPC circulating tumor PC, NPC normal PC, SP solitary plasmacytoma, macrofocalMM macrofocal MM, MGUS monoclonal gammopathy of undetermined significance, SMM smoldering MM, MM multiple myeloma, PB peripheral blood, NGF next-generation flow, HD healthy donors

Most PCN patients -185/264 (70%)- showed CTPC in PB. The frequency of cases in which CTPC were detected in PB progressively increased (*p* <0.05) from SP patients (18%) and macrofocalMM (25%), to MGUS (59%), and both SMM (100%) and MM (100%) cases (Fig. 1a). In parallel, progressively higher numbers (*p* *<*0.05) of CTPC in PB were found from SP and macrofocalMM patients (median in both groups: <0.001 CTPC/μL) to MGUS (median: 0.008 CTPC/μL), SMM (median: 0.16 CTPC/μL) and MM (1.9 CTPC/μL)- (Fig. 1b and Supplemental Table 4).

In turn, NPC were detected in PB of all healthy donors (median: 1.9 NPC/µL) and, at lower (*p* <0.001) numbers, also in all PCN patients (median: 1.0 NPC/µL). In more detail, significantly decreased NPC counts were found in PB of MGUS (*p* <0.001) and SMM (*p* = 0.01), but not in SP, macrofocalMM and MM cases who had normal (*p*> 0.05 vs. HD) NPC levels (Fig. 1c and Supplemental Table 5). This altered distribution of PB CTPC and NPC translated into a progressively increased median percentage of CTPC within the whole PB PC compartment from SP and macrofocalMM (0%) to MGUS (0.8%), SMM (15.8%), and MM (47.9%) (*p* < 0.05; Fig. 1d and Supplemental Table 4).

**Fig. 2:**
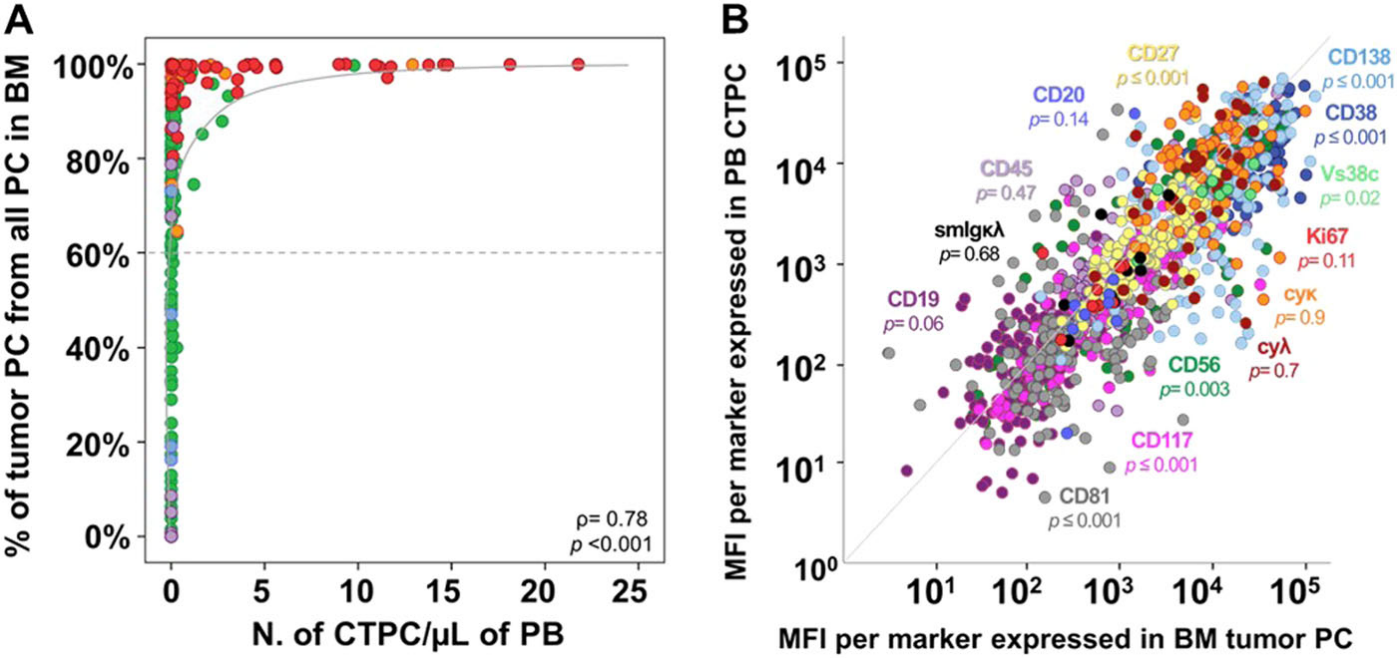
Correlation between the number and immunophenotype of tumor PCs in paired PB and BM samples from newly diagnosed PCN patients. Correlation between the percentage of TPC from all BMPC and the absolute PB CTPC counts in paired BM and PB samples are shown in **a**, while the correlation between median fluorescence intensity (MFI) levels of expression of individual phenotypic markers in paired BM TPC vs. PB CTPC are displayed in **b**. In **a**, dots are colored per diagnostic category as follows: SP patients are color-coded as purple circles, macrofocalMM as light blue circles, MGUS cases are represented as green circles, SMM as orange circles, and MM as red circles. The dotted line represents the percentage of BM TPC above, which CTPC are usually detected in PB of PCN patients (91% vs. 9% cases with CTPC were found for patients above and below the line, respectively). In **b**, individual phenotypic markers are color-coded as follows: CD38, dark blue; CD56, dark green; CD45, light purple; CD19, dark purple; CD117, pink; CD81, gray; CD138, light blue; CD27, yellow; CyKappa, orange; CyLambda; brown; Vs38c, light green; SmKappa + SmLambda, black; Ki67, red; and, CD20, blue. PB peripheral blood, BM bone marrow, PC plasma cell, TPC tumor PC, CTPC circulating tumor PC, NPC normal PC, PCN PC neoplasms, MFI median fluorescence intensity, SP solitary plasmacytoma, macrofocalMM macrofocal MM, MGUS monoclonal gammopathy of undetermined significance, SMM smoldering MM, MM multiple myeloma

Of note, a strong (non-linear) correlation was observed between the percentage of TPC from all BMPC and the absolute number of PB CTPC in paired (PB and BM) samples (*ρ* = 0.78; *p* *<* 0.001), TPC typically becoming detectable in PB when they represented ≥ 60% of the whole BMPC compartment (Fig. 2a). Of note, such correlation remained significant even when patients with localized disease (SP and macrofocalMM) (*ρ* = 0.54, *p* = 0.02), MGUS (*ρ* = 0.64, *p*< 0.0001), SMM (*ρ* = 0.51, *p* = 0.02) and MM (*ρ* = 0.55, *p* <0.0001), were analyzed separately.

From the phenotypic point of view, although PB CTPC showed a similar profile to that of BM TPC, they displayed significantly lower (*p* *<* 0.05) expression levels of the CD38, CD138, CD81, CD56, CD27, and Vs38c maturation-associated markers, together with CD117 and to a lesser extent also the Ki67-proliferation marker (*p* = 0.11), supporting a more immature and less proliferative immunophenotype for paired PB vs. BM TPCs. Other maturation-associated PC markers displayed either a tendency towards lower (CD20, *p* = 0.14; and CD19, *p* = 0.06), or similar expression levels -CD45 (*p* = 0.47) and Sm/CyIg light chains (SmIgκ/λ, *p* = 0.68; CyIgκ, *p* = 0.9; CyIgλ, *p* = 0.7)- in BM vs. PB TPC (Fig. 2b and Supplemental Fig. 1).

### Association between the number of PB CTPC and the distinct diagnostic and prognostic categories of the disease

**Fig. 3:**
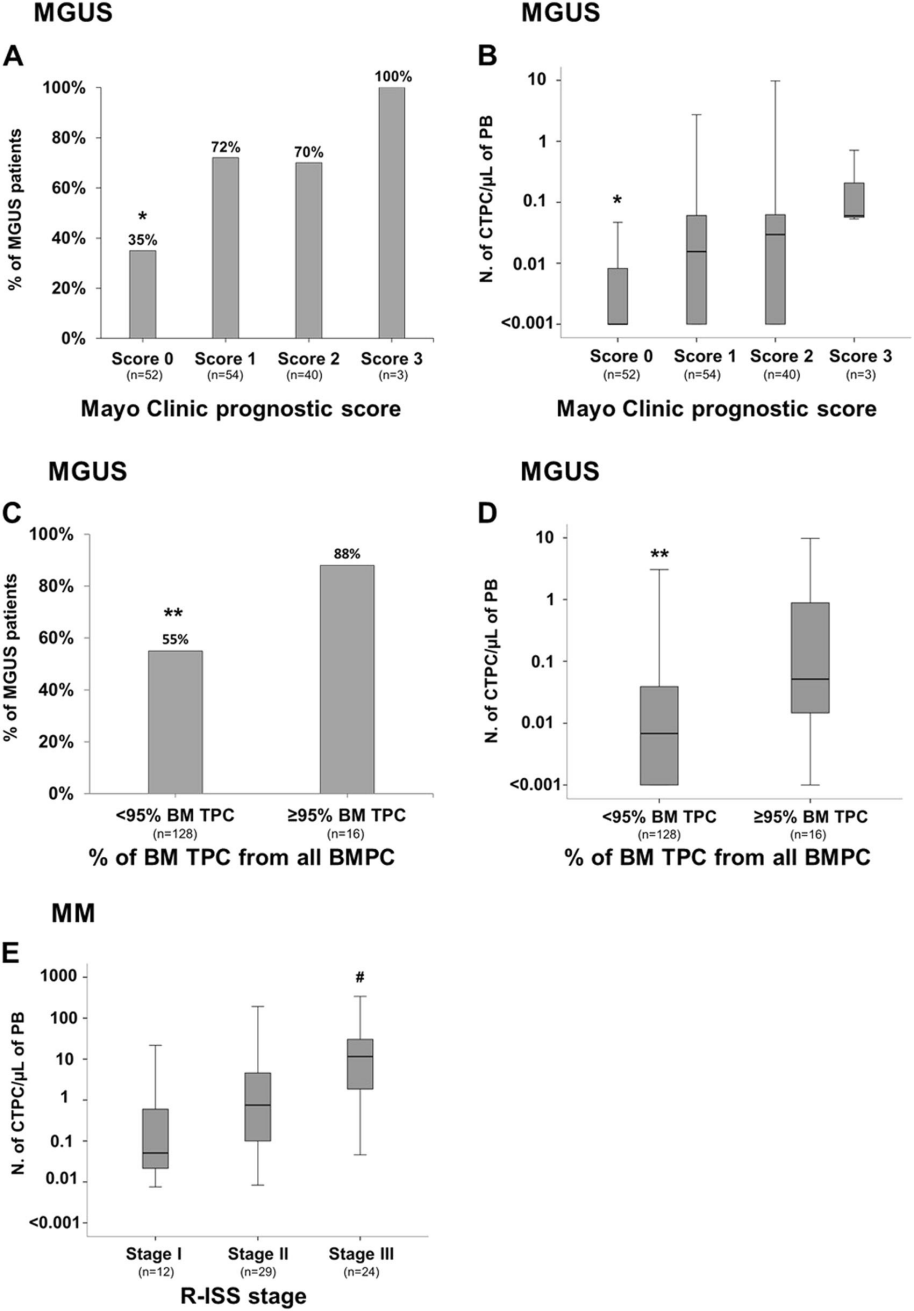
Frequency and distribution of circulating tumor PC in PB of MGUS and MM patients classified into distinct risk-groups and clinical stages, respectively. Frequency of MGUS patients presenting with CTPC and their absolute counts according to the Mayo Clinic prognostic index (**a** and **b**, respectively; *p < 0.05 for Mayo Clinic prognostic score 0 vs. scores 1, 2, and 3) and the distribution of TPC within the whole BMPC compartment (<95% vs. ≥ 95%) (**c** and **d**, respectively; **p < 0.05 vs. ≥ 95% TPC from all BM PC). In **e**, the absolute counts of PB TPC in MM patients distributed according to the R-ISS stages is shown (^#^*p* < 0.05 for stage III vs. stages I and II). Boxes extend from the 25th to the 75th percentile values; the line in the middle and vertical lines correspond to the median value and the 10th and 90th percentiles, respectively. PC plasma cell, TPC tumor PC, CTPC circulating tumor PC, PB peripheral blood, BM bone marrow, MGUS monoclonal gammopathy of undetermined significance, MM multiple myeloma, R-ISS revised international staging system

**Fig. 4:**
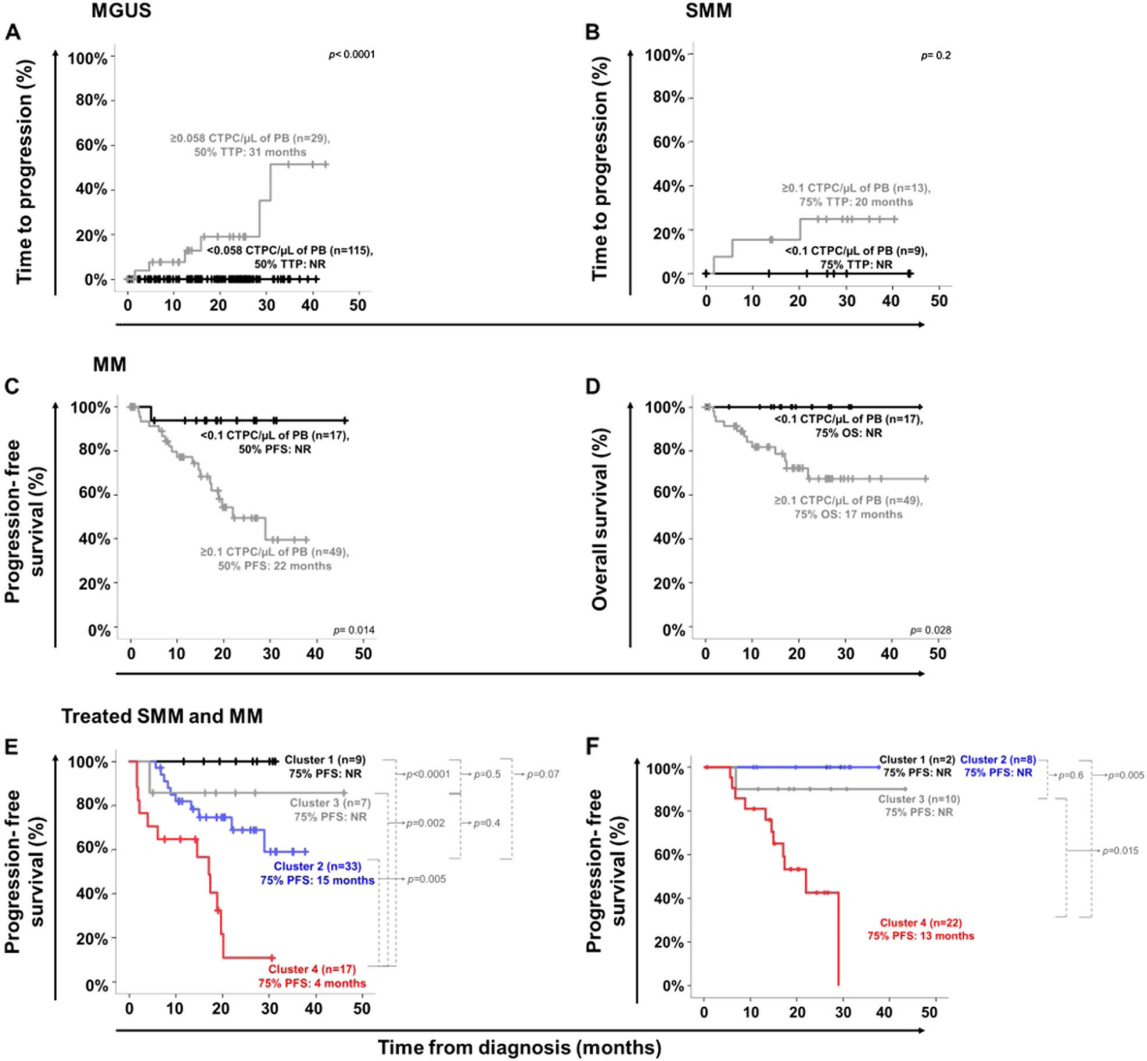
Impact of PB CTPC counts at diagnosis on the outcome of MGUS, SMM, and MM patients. TTP curves of MGUS and SMM grouped according to the absolute number of PB CTPC are shown in **a** and **b**, respectively. **c** and **d** display PFS and OS curves of MM patients grouped according to the absolute number of CTPC per μL of PB. In **e** and **f** PFS curves of treated myeloma (SMM and MM) patients grouped according to the absolute count of CTPC in PB detected at diagnosis and either the standard IMWG response criteria (**e**) or the BM MRD status (**f**) reached after therapy, are displayed, respectively. Patients who reached VGPR, CR/sCR or BM MRD-negativity after therapy and had low (black line) vs. high (blue line) levels of PB CTPC at diagnosis, are included in clusters 1 and 2 from both **e** and **f**, respectively; in turn, patients who did not reach VGPR, CR/sCR (e.g., PR, SD or PD) or BM MRD-negativity (e.g., BM MRD-positive cases) after therapy and had low (gray line) vs. high (red line) PB CTPC counts at diagnosis, are included in clusters 3 and 4 in **e** and **f**, respectively. TTP time to progression, PFS progression-free survival, OS overall survival, PC plasma cell, CTPC circulating tumor PC, NR not reached, PB peripheral blood MGUS monoclonal gammopathy of undetermined significance, SMM smoldering MM, MM symptomatic multiple myeloma, IMWG International Myeloma Working Group, VGPR very good partial response, CR complete response, sCR stringent complete response, PR partial response, SD stable disease, PD progressive disease

ROC curve analysis showed that the most accurate (88% accuracy) cutoff to discriminate between MGUS and MM was at a PB count ≥ 0.058 CTPC/μL (*p* <0.001) (Table 1). Within MGUS, cases at very low-risk of malignant transformation as defined by both the Mayo Clinic index (i.e., score 0) and the Spanish criteria (a percentage of TPC within the total BMPC of <95%), less commonly showed CTPC in PB: 35% vs. 72%, 70% and 100% for the Mayo Clinic scores 1, 2, and 3, respectively (*p* ≤ 0.03; Fig. 3a), and 55% vs. 88% for MGUS cases with <95% vs. ≥ 95% TPC/all BMPC (*p* *=* 0.01; Fig. 3c), respectively. Likewise, the number of CTPC also increased significantly from MGUS cases with Mayo Clinic score 0 to score 1, 2, and 3 cases (*p* ≤ 0.003; Fig. 3b) and in MGUS cases with ≥ 95% vs. <95% TPC/all BMPC (*p* = 0.001; Fig. 3d). Interestingly, despite the still limited follow-up, those MGUS cases with higher absolute PB CTPC counts showed significantly greater (*p* *<*0.0001) rates of progression to SMM and MM -6/29 (21%) MGUS cases had progressed to SMM (*n* = 1) and MM (*n* = 5) at 30 months- compared to MGUS cases who showed low or undetectable CTPC in PB -0/115 cases (0%)- (Table 1 and Fig. 4a).

**Table 1:** Most accurate cutoff to discriminate between MM and MGUS cases based on the absolute number of PB circulating tumor PC

Variable	No. of CTPC/μL of PB
Cutoff value	0.058 CTPC/μL
Sensitivity	80%
Specificity	80%
AUC	88%
Positive predictive value (%)	65%
Negative predictive value (%)	90%
No. of MGUS cases below cutoff/total (%)	120/150 (80%)**
No. of MM cases above cutoff/total (%)	55/68 (81%)
False-positive cases (%)	30/150 (20%)
False-negative cases (%)	13/68 (19%)

*PB* peripheral blood, *PC* plasma cell, *CTPC* circulating tumor PC, *MGUS* monoclonal gammopathy of undetermined significance, *MM* symptomatic multiple myeloma, *AUC* area under the curve, *No.* number.

*p *<*0.0001. **6/30 (20%) MGUS cases above the cutoff have progressed to MM after a median follow-up of 17 months

In contrast to MGUS, no significant differences in PB CTPC counts were observed among SMM patients classified into different risk-groups by the Mayo Clinic prognostic index (*p* = 0.2; Supplemental Fig. 2a) and the Spanish score (*p* = 0.5; Supplemental Fig. 2b), which could be due to the limited number of high-risk cases (score 2) analyzed and/or the independent value of the two parameters. Despite this, at nearly 2 years, ~25% of SMM with higher numbers of CTPC in PB ( ≥ 0.1 CTPC/μL) had progressed to active MM vs. 0% among SMM patients with lower PB CTPC counts, although differences did not reach statistical significance (*p* = 0.2) (Fig. 4b).

Among MM, R-ISS stage III cases showed significantly higher counts (Fig. 3e) of CTPC in PB (*p* = 0.001 and *p* = 0.004 vs. stage I and stage II cases, respectively). Interestingly, MM cases with low numbers (i.e., <0.1 CTPC/µL of PB- that would correspond to an MGUS-like pattern) of CTPC, showed prolonged 2 years PFS and OS rates (Figs. 4c, d, respectively): PFS of 94% vs. 40% (*p* = 0.014) and OS of 100% vs. 67% (*p* = 0.03), respectively-. Surprisingly, the longer PFS rates of MM cases who showed lower numbers of CTPC in PB was independent of response to therapy both when the IMWG complete response (CR) status (*p* <0.0001; Fig. 4e) and the BM MRD status (*p* = 0.02; Fig. 4f) were considered.

## Discussion

In the past, progressively higher frequencies of MGUS and MM patients presenting with CTPC in PB have been reported in parallel to an increased sensitivity of the techniques used (e.g., immunocytochemistry vs. conventional 4- and 8- color flow)^19,20,22–24,29,47,48^. Here, we applied for the first time the recently described high-sensitive NGF method^34^ for the detection of CTPC in PB of a large cohort of newly diagnosed MGUS, SMM and MM cases, including also for the first time, SP and macrofocalMM patients. Overall, our results showed an up to ~2-fold increased frequency of cases presenting with CTPC in PB by NGF vs. both immunocytochemistry and conventional flow cytometry, among MGUS (59% vs. 19–37%)^19,25^, SMM (100% vs. 15–50%)^29,49^ and MM (100% vs. 50–73%)^19,25,26^. In contrast, only a small percentage of SP and macrofocalMM had detectable CTPC in PB. Altogether, these results confirm and extend on previous observations indicating that the presence of CTPC in PB is usually associated with systemic disease (i.e., MGUS, SMM and MM), higher numbers of PB CTPC within patients with systemic disease reflecting a more malignant clinical behavior^19,22,50^, while it is a rare finding among tissue-localized PC tumors (e.g., SP and macrofocalMM)^14^. In line with these findings, the overall number of PB CTPC as assessed by NGF also increased progressively from SP and macrofocalMM to MGUS, SMM, and MM, the number instead of the presence vs. absence of CTPC providing an accurate discrimination between MGUS and MM in the great majority of patients. Altogether, these findings would support further evaluation of the benefit of including PB CTPC counts in new minimally invasive (i.e., PB-based) diagnostic algorithms, to distinguish between MGUS and MM; alternatively, it might be used as a prognostic factor in both diseases, as discussed below. Although next-generation sequencing approaches were not explored in parallel to NGF in our cases, previous reports from the literature suggest a similar sensitivity (i.e., detection of 1 CTPC in 10^6^ total cells; 0.0001%) but a slightly lower applicability (*n* = 44/46 patients; 96%)^51,52^.

Previous studies based on less sensitive approaches indicated that the presence of CTPC in PB and/or their number, are both associated with (i) an increased risk of transformation of MGUS to MM^20,23^ and (ii) the outcome of SMM and MM, when assessed both at diagnosis^22,29,47,48,53^ and after therapy^21,31–33^. Despite the still relatively limited number of patients investigated per diagnostic category and the short median follow-up, our results confirm and extend on these findings. Thus, MGUS showing higher numbers of PB CTPC displayed shorter TTP to MM, while in SMM, the prognostic impact of the number of CTPC in PB appears to be more limited and independent from both the Mayo Clinic and the Spanish scoring systems. Nevertheless, the data on SMM should be interpreted with caution due to the limited number of these cases. Most interestingly, CTPC counts in MM within the range of MGUS patients were associated for the first time here, with a significantly longer PFS and OS, independently of response to therapy evaluated according to both the CR and MRD status. Altogether, these results reinforce the notion that also within MM, the presence of high number of CTPC is a strong adverse prognostic factor, very low numbers of CTPC in PB at diagnosis (i.e., similar to those observed in MGUS), potentially contributing to the identification of those few MM cases that show a good long-term outcome, even when they do not reach BM MRD-negativity or CR. Of note, preliminary results from our group further show that the persistence/presence of CTPC in MM patients who had undergone therapy, might be used as a surrogate marker of BM MRD-positivity, since all treated MM patients who showed CTPC after therapy, always showed MRD + of paired BM samples (data not shown). Confirmation of these findings deserves further prospective studies in large series of patients with longer follow-up.

The precise biological significance of the presence and the levels of CTPC in PB of MGUS and MM patients, still remains largely unknow^25,54^. Classically, the presence of CTPC in PB of MM has been viewed as a sign of dissemination of BM TPC into the circulation leading to distinct tissue-homing patterns and the formation of new PC tumors at distant (e.g., extramedullary) sites^19,25,55^. However, recent studies show that compared to BM TPC, PB CTPC display features of more quiescent cells with greater resistance to chemotherapeutic agents and higher potential for self-renewal, together with a potentially more immature phenotype^19,25,56,57^, suggesting that PB CTPC might constitute (and behave as) true MM stem cells^55,58,59^.

Here, we confirm that PB CTPC from MM and MGUS patients are immunophenotypically more immature than their BM counterpart, as reflected by the expression of significantly lower levels of (i) markers that are typically acquired by PC during migration from secondary lymphoid tissues to the BM, such as CD38 and CD138^60–64^, and (ii) adhesion molecules that anchor PC to stromal structures such as CD56, CD81, and CD117^60,65–67^. Of note, here we also show that PB CTPC display lower expression levels of activation/differentiation-associated antigens such as CD27^67^ and Vs38c, a rough endoplasmic reticulum protein directly linked to a high rate of protein (i.e., Ig) synthesis and secretion^64,68,69^. In line with previous observations, PB CTPC detected in this large series of patients also tended to show lower levels of expression (vs. BMPC) of the Ki67-proliferation associated marker^9,25,55,56^. In contrast, we did not found significant differences as regards the phenotypic profile of PB vs. BM TPC for other maturation-associated antigens previously described to be aberrantly expressed by TPC in MM and MGUS patients, such as CD19, CD20, CD45 and sm/cyIg;^49,62^ this might be due to the fact that the pattern of expression of these markers could more closely reflect tumor phenotypes potentially associated with specific genetic lesions -e.g., CD20 expression in cases carrying *t*(11;14)-^70^ than actual maturation-associated phenotypes. Despite all the above, CTPC in MM might also correspond to an admixture of immature (i.e., potential stem cell-like) TPC and more mature (i.e., BM derived) myeloma PC. In any case, if this whole concept about the greater immaturity and stem cellness of PB vs. BM TPC holds true, PB CTPC in MM and MGUS might play a key role in disease dissemination throughout the BM (and in a subset of MM patients, also to extramedullary sites), at the same time they would be unable to appropriately home in the BM when niches are (almost) full and occupied by long-living (more mature) and growing tumor PCs. Altogether, this might also contribute to explain, at least in part, the non-linear (significant) correlation here reported between the PB and BM TPC burden. Similarly, it might also contribute to explain the unexpectedly higher number of circulating NPC in PB in more advanced vs. earlier stages of the disease (i.e., MM vs. MGUS), despite an almost complete depletion of their normal long-living BMPC counterpart is frequently observed in association with low serum non-involved immunoglobulin levels in MM, but not in MGUS. In such case, progressive unspecific blockade of both TPC and (recently produced short-lived) NPC into the BM, due to the lack of (empty) PC niches occupied by TPC would occur together with a parallel increase in PB of both CTPC and NPC. The long-living nature of CTPC would provide an advantage to this PC population over short-lived NPC (recently produced in lymphoid tissues). This would lead to selective accumulation of TPC in the BM with progressive depletion of normal long-living PC in BM. As a consequence, abnormally low serum antibody production and immuneparesis would emerge as a hallmark of advanced disease^43,71^.

In summary, here we show that the presence of CTPC in PB as assessed by NGF is a hallmark of both SMM and MM and a highly frequent finding among MGUS, while absent in most SP and macrofocalMM cases. Higher numbers of CTPC in PB were strongly associated with features of malignant disease, providing a powerful minimally-invasive blood test to discriminate between MGUS and MM at diagnosis and to identify both (i) MGUS cases at high-risk of progression to MM, and (ii) a small subset of MM patients with low number of CTPC (within the range of MGUS cases) that display a significantly longer survival despite not achieving BM MRD-negativity or CR.

## Disclaimer

The EuroFlow consortium is an independent scientific consortium, which aims at innovation and standardization of diagnostic flow cytometry. All acquired knowledge and experience within EuroFlow is shared with the scientific and diagnostic community after protection of the relevant Intellectual Property, for example by filling patents. The involved patents are owned by the EuroFlow Consortium and licensed to companies, including Cytognos SL (Salamanca, Spain), Becton/Dickinson Biosciences (San José, CA, USA), and Immunostep SL (Salamanca, Spain). The revenues of the patents are exclusively used for EuroFlow Consortium activities, such as for covering (in part) the costs of the Consortium meetings, the EuroFlow Educational Workshops and the purchase of custom-made reagents for collective experiments. F.M.J., v.D.J.J.M., and O.A. are part of the inventors on the EuroFlow-owned patent PCT/NL/2013/050420; US 62/072,498 (Methods, reagents and kits for detecting minimal residual disease). This patent is licensed to Cytognos, which pays royalties to the EuroFlow Consortium

## Electronic supplementary material

Supplemental Material

Supplemental Table 1

Supplemental Table 2

Supplemental Table 3

Supplemental Table 4

Supplemental Table 5

Supplemental Figure 1

Supplemental Figure 2
