# Supplementary material for: Next generation flow for minimally-invasive blood characterization of MGUS and multiple myeloma at diagnosis based on circulating tumor plasma cells (CTPC)
Source: Blood Cancer J. 2018 Nov 19;8(12):117. doi: 10.1038/s41408-018-0153-9 (PMC6242818; doi:10.1038/s41408-018-0153-9)
Supplement: Supplementary file 1 — Supplemental Material [file 41408_2018_153_MOESM1_ESM.docx]

**SUPPLEMENTAL MATERIALS:**

**Title:** Next generation flow for minimally-invasive blood characterization of MGUS and multiple myeloma at diagnosis based on circulating tumor plasma cells (CTPC)

**Authors:** Sanoja-Flores L^1^*, Flores-Montero J^1^*, Garcés JJ^2^*, Paiva B^2^*, Puig N^3^, García-Mateo A^4^, García-Sánchez O^3^, Corral-Mateos A^1^, Burgos L^2^, Blanco E^1^_,_ Hernández-Martín J^4^, Pontes R^5^, Diez-Campelo M^3^, Millacoy P^6^, Rodríguez-Otero P^2^, Prosper F^2^, Merino J^2^, Vidriales MB^3^, García-Sanz R^3^, Romero A^7^, Palomera L^8^, Ríos-Tamayo R^9^, Pérez-Andrés M^1^, Blanco JF^10^, González M^3^, van Dongen JJM^11^, Durie B^12^, Mateos MV^3^, San-Miguel J^2^ and Orfao A^1^.

*These authors have equally contributed to this paper and should be considered as first authors.

**Affiliations:**

1. Cancer Research Center (IBMCC-CSIC/USAL-IBSAL); Cytometry Service (NUCLEUS) and Department of Medicine, University of Salamanca, Salamanca, Spain (USAL). Centro de Investigación Biomédica en Red de Cáncer, Instituto Carlos III, Madrid, Spain. CIBER-ONC number CB16/12/00400.
2. Clinica Universidad de Navarra; Applied Medical Research Center (CIMA), IDISNA, Pamplona, Spain (UNAV). CIBER-ONC number CB16/12/00369 and CB16/12/00489.
3. Department of Hematology, University Hospital of Salamanca, IBSAL; IBMCC (USAL-CSIC), Salamanca, Spain (HUSA). CIBER-ONC number CB16/12/00233.
4. Department of Hematology, Health Care Center of Segovia, Segovia, Spain (CAS).
5. Faculty of Medicine, Federal University of Rio de Janeiro and Institute of Pediatrics and Childhood Care, Rio de Janeiro, Brazil.
6. Department of Hematology, Hospital Center of Navarra (CHN), Pamplona, Spain.
7. Primary Care Center Miguel Armijo, Sanidad de Castilla y León (SACYL), Salamanca, Spain.
8. Department of Hematology, University Hospital Lozano Blesa, Zaragoza, Spain (HULB).
9. Department of Hematology, Virgen de las Nieves Hospital, Granada, Spain (HVN).
10. Departament of Orthopedics, University Hospital of Salamanca, IBSAL; IBMCC (USAL-CSIC), Salamanca, Spain.
11. Department of Immunohematology and Blood Transfusion, Leiden University Medical Center, Leiden, The Netherlands.
12. Cedars-Sinai Samuel Oschin Cancer Center, Los Angeles, CA, USA.

**PATIENTS AND METHODS**

**Immunophenotypic detection of circulating tumor plasma cells (CTPC).** Briefly, peripheral blood (PB) (median volume of 5.1 mL; range: 2.1-12.8 mL) was mixed with 50 mL of an ammonium chloride (NH_4_CL) lysing solution and incubated for 15 minutes at room temperature (RT) to lyse non-nucleated red cells. Then, nucleated cells were washed in phosphate buffered saline (PBS) containing 0.5% bovine serum albumin (BSA) and 0.09% sodium azide (NaN_3_); for surface membrane (Sm) staining only, washed cells were subsequently stained with the different antibodies for 30 minutes at RT in the darkness. Afterward, 2 mL of the FACS lysing solution -Becton/Dickinson Biosciences (BD), San Jose, CA,- was added to the cell pellet, mixed, and another incubation was performed for 10 minutes (RT). Identification of cytoplasmic immunoglobulins (CyIg) was performed according to the EuroFlow standard operating procedure for simultaneous staining of cell surface membrane (Sm) and cytoplasmic (Cy) markers using the Fix&Perm reagent kit (An der Grub, Vienna, Austria) (for more detailed protocol information please see [www.EuroFlow.org](http://www.euroflow.org/)). In tube 1 of the EuroFlow-IMF MM minimal residual disease (MRD) antibody panel^34^, cells were stained for: CD138-BV421, CD27-BV510, CD38ME-FITC, CD56-PE, CD45-PerCPCy5.5, CD19-PECy7, CD117-APC and CD81-APCC750; while in tube 2, the CD138-BV421, CD27-BV510, CD38-FITC, CD56-PE, CD45-PerCPCy5.5, CD19-PECy7, Cy-IgKappa (κ)-APC and CyIgLambda (λ)-APCC750 stainings, were used (Supplemental Table 3).

**LEGENDS TO SUPPLEMENTAL FIGURES:**

**Supplemental Figure 1. Illustrating example of the immunophenotypic features of normal PC (blue dots) and tumor PC (red dots) from paired BM (Panel A and B) and PB (Panel C and D) samples from a representative newly-diagnosed MM patient.** Principal Component 1 (PC) vs PC2 analysis of the overall immunophenotype of normal (blue dots) vs tumor (red dots) PC in paired BM (Panel A) and PB (Panel C) samples for the 8-markers included in tube 1 plus CyIgKappa/CyIgLambda (tube 2) of the NGF EuroFlow-IMF MM MRD antibody panel. In the right panels, conventional bidimensional dot plots illustrating the specific phenotypic and light scatter patterns observed for BM (Panel B) and PB (Panel D) normal (blue dots) and tumor (red dots) PC are shown. PB, peripheral blood; BM, bone marrow; PC, plasma cell; MM, multiple myeloma; CyIg, cytoplasmic immunoglobulin; NGF, next generation flow; IMF; International Myeloma Foundation; MRD, minimal residual disease.

**Supplemental Figure 2. Absolute CTPC counts in PB of SMM patients grouped according to the Mayo Clinic prognostic index (Panel A) and the Spanish prognostic score (Panel B).** Boxes extend from the 25^th^ to the 75^th^ percentile values; the line in the middle and vertical lines correspond to the median value and the 10^th^ and 90^th^ percentiles, respectively. PC, plasma cell; CTPC; circulating tumor PC; PB, peripheral blood; SMM, smoldering multiple myeloma.
