# Supplementary figures and images for: Next generation flow for minimally-invasive blood characterization of MGUS and multiple myeloma at diagnosis based on circulating tumor plasma cells (CTPC)
Source: Blood Cancer J. 2018 Nov 19;8(12):117. doi: 10.1038/s41408-018-0153-9 (PMC6242818; doi:10.1038/s41408-018-0153-9)

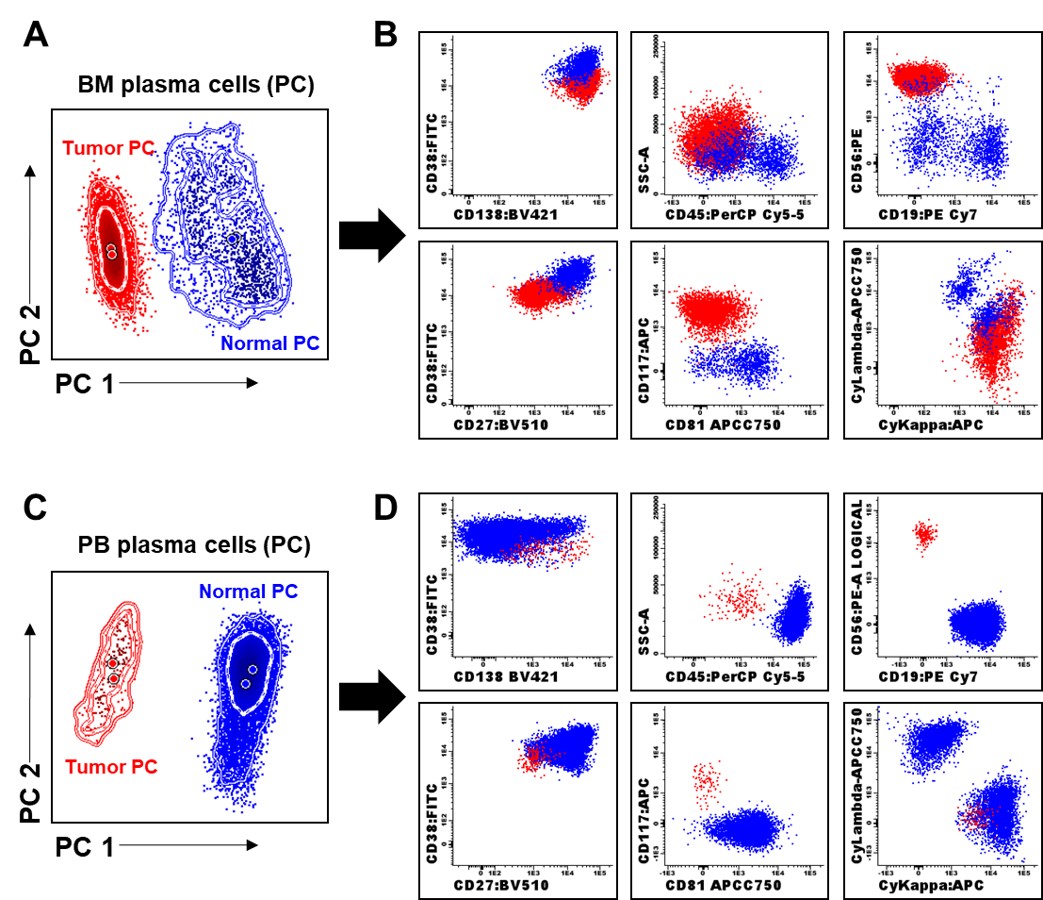

Supplement: Supplementary file 7 — Supplemental Figure 1 [file 41408_2018_153_MOESM7_ESM.jpg]

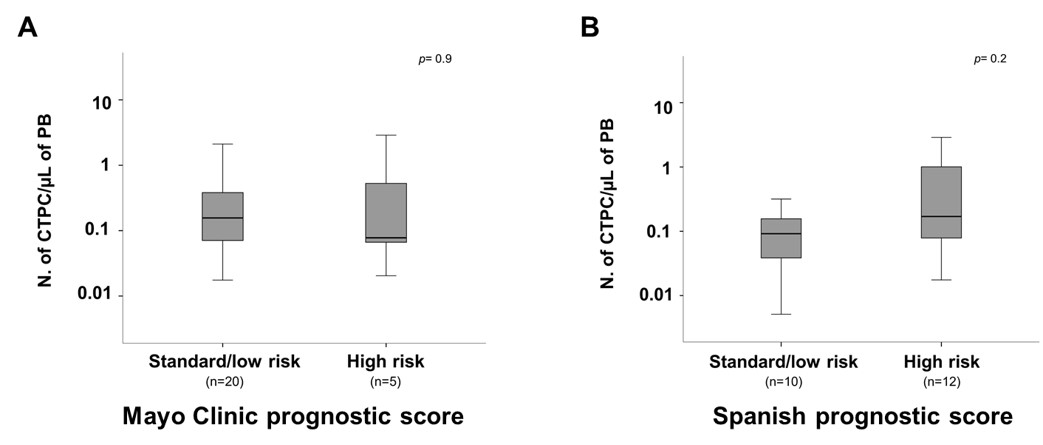

Supplement: Supplementary file 8 — Supplemental Figure 2 [file 41408_2018_153_MOESM8_ESM.jpg]
